# Healthy lifestyle in low-socioeconomic groups: expert views

**DOI:** 10.3389/fpubh.2025.1511317

**Published:** 2025-05-21

**Authors:** Liza A. Hoveling, Manna A. Alma, Jeanet A. Landsman-Dijkstra, Nynke Smidt, Sijmen A. Reijneveld, Ute Bültmann, Marlou L. A. De Kroon

**Affiliations:** ^1^Department of Epidemiology, University Medical Center Groningen, University of Groningen, Groningen, Netherlands; ^2^Department of Health Sciences, Applied Health Research, University Medical Center Groningen, University of Groningen, Groningen, Netherlands; ^3^Department of Health Sciences, Community and Occupational Medicine, University Medical Center Groningen, University of Groningen, Groningen, Netherlands; ^4^Department of Public Health and Primary Care, Environment and Health, KU Leuven, Leuven, Belgium

**Keywords:** health promotion, socioeconomic factors, lifestyle, focus groups, semistructured interview

## Abstract

**Background:**

Low socioeconomic position (SEP) increases the risk of health inequalities. Understanding how to realize lifestyle improvements in groups with low SEP may offer routes to reduce these inequalities, and information from professionals involved in this target can support this. Our aims were to gain insight into the professionals’ perspectives on factors that influence the lifestyle of individuals with low SEP, and to identify promising approaches to reduce health inequalities.

**Methods:**

Focus group interviews and semi-structured interviews were conducted with 28 social workers (partially in training), health care and policy professionals. All interviews were audio recorded, transcribed verbatim, and analysed following thematic analysis.

**Results:**

Four themes emerged from the data: individual factors, accumulating problems, social context and societal factors; for the second aim a fifth theme emerged: involvement of individuals with low SEP. Participants also indicated a hierarchy in these themes. Addressing accumulating multi-faceted issues such as poverty is imperative before a more healthy lifestyle can be adopted and individuals can focus on skills needed to achieve a healthy lifestyle. Additionally, the social context (e.g., parents or neighbours) can play a supportive role in lifestyle change, supported by government and with active involvement of low SEP individuals.

**Conclusion:**

According to the professionals, to help individuals with low SEP to realize a healthier lifestyle, poverty, knowledge and skills, and the involvement of a person’s social context and society should be addressed.

## Introduction

Socioeconomic inequalities in many health outcomes are large and persistent (1). Individuals with low socioeconomic position (SEP) often have a more unhealthy lifestyle than their higher SEP counterparts; they more often smoke, have an unhealthier diet, have worse sleeping habits, and are less physically active during leisure time (2, 3). An unhealthy lifestyle is in turn related to both adverse physical and mental health outcomes (4, 5). Given that lifestyle is potentially modifiable, it is important to understand how to improve lifestyle in individuals with low SEP in order to reduce SEP inequalities.

Several factors contribute to the unhealthy lifestyles of individuals with low SEP, including specific attitudes, non-supportive social norms, and insufficient self-efficacy, beliefs, and skills to maintain a healthy lifestyle (6–9). Low SEP individuals may feel less inclined to seek lifestyle advice due to challenges in their daily life, such as financial struggles and relationship issues (10–13). They also rely more on social support for making healthy choices (10). Upstream factors, like immediate crises related to their socioeconomic status, often take precedence over long-term health concerns (14). Understanding both upstream and downstream factors is crucial for addressing lifestyle changes in this group. Despite previous intervention efforts, reducing health inequalities has been challenging (15–17). Evidence from qualitative research may help to obtain a comprehensive understanding of why low SEP individuals lead unhealthier lives and how they can be supported in adopting healthier lifestyles.

The Social Determinants of Health model provides a framework for understanding the broader structural influences on lifestyle behaviors in individuals with a low SEP. The model highlights how factors such as income, education, employment, housing, and access to healthcare shape health outcomes and contribute to persistent health inequalities (18). Research has shown that these determinants influence lifestyle choices, as limited resources, adverse living conditions and health literacy can create barriers to healthy behaviors (19, 20). Addressing these determinants is pivotal for developing effective health promotion strategies that go beyond individual behavior change and incorporate systemic and policy-level interventions. (19, 20).

Reaching individuals with low SEP and supporting a healthier lifestyle may be more successful by tailored approaches (21). Several studies showed that, instead of following the standard principles of health promotion, attention should be paid to the capacities (e.g., abilities, resources and potential) of individuals with low SEP (12, 21–24). Furthermore, their social context should also be (more) involved to help them make lifestyle changes (8, 10, 11, 25–27). To achieve change it is important to understand which factors influence lifestyle, and how professionals closely involved with these individuals at neighbourhood level believe that change should be approached based on their personal experiences with the target group.

This study aims to explore professionals’ perspectives on implementing lifestyle promotion for low SEP individuals, on which evidence is scarce as yet. These professionals possess valuable expertise in this field. The qualitative study seeks insights from social workers, trainee social workers, healthcare, and policy professionals on (1) lifestyle influencers in low SEP individuals and (2) effective strategies for promoting healthy lifestyles in this group.

## Methods

A qualitative exploratory research design, grounded in a constructivist paradigm (28), was used. This paradigm assumes that reality is socially constructed and shaped by interactions and experiences, making it suitable for exploring professionals’ perspectives on lifestyle promotion among low SEP individuals. In this qualitative study we used semi-structured interviews and focus group interviews to explore the views of professionals regarding factors influencing lifestyle, and potential preventive approaches to offer to individuals with low SEP. We used the Consolidated Criteria for Reporting Qualitative Health Research (COREQ) (29) (see Supplementary Table 1).

### Study context

Our study was conducted in the Netherlands, where socioeconomic health inequalities remain a persistent challenge, despite a relatively well-developed social security system. Individuals with low SEP often experience multiple disadvantages, including financial instability, limited access to healthcare, and higher exposure to unhealthy living conditions (30). Dutch public health policies emphasize preventive care and lifestyle interventions, yet reaching and effectively supporting low SEP groups remains difficult (31–33). Professionals working in healthcare, social work, and policymaking play a role in bridging this gap by implementing tailored lifestyle interventions. Their perspectives provide valuable insights into the structural and behavioral challenges that low SEP individuals face, as well as effective strategies for promoting healthier lifestyles in this population (30–33).

### Participants

We aimed at including professionals who work with low SEP individuals as proxy informants, considering the COVID-19 crisis during our recruitment period to block inclusion of low SEP individuals. These professionals have proximity to and insight in the target group and on top can also help bridge the gap between practice and research, making them key figures in translating insights from practice to science. Participants were recruited for one-time involvement through the networks of the Collaborative Research Center on Public Health in the Northern Netherlands, and through networks of the researchers involved in this study. To ensure rigor in participant selection, we employed a purposeful sample strategy, aiming for maximum variation in professional background, experience level, and roles in working with low SEP individuals. We included four types of professionals: social workers (*n* = 3), social workers in training, partially also coming from low SEP households themselves (*n* = 9), health care professionals (*n* = 7), and policy professionals (*n* = 9). Table 1 presents key characteristics of the participants. All included participants had work activities related to and contact with individuals with low SEP, often living in poverty. Ethical approval for the study was obtained from the Medical Ethics Committee of the University Medical Center Groningen. Participants received written information about the procedure and a short questionnaire on their age, sex, employer, occupation, and their low SEP target group (children, adolescents, adults, or the older adults). All recruited individuals participated in the study. We ensured ethical integrity by obtaining written informed consent from all participants, safeguarding confidentiality through anonymized data handling, and adhering to the Helsinki standards on health research. All participants gave written informed consent and received a gift voucher of 20 euros for their participation.

**Table 1:** Key characteristics of study participants.

Professional group	*n* (%)	Average age (years)	Female (%)	Average years of experience
Social workers	3 (11%)	32.7	66.6%	8.4
Social workers in training	9 (32%)	17.0	66.6%	1.2
Health care professionals	7 (25%)	48.3	100%	15.6
Policy professionals	9 (32%)	38.7	88.9%	11.1
Total	28 (100%)		78.6%	9.3

### Data collection

We collected data in three phases using an iterative process, meaning that information from the previous phase was carried over into the next phase. First, we conducted a semi-structured interview with a policy professional, and a focus group interview with social workers in training. Second, we conducted focus group interviews with health care professionals and policy professionals. Third, we conducted semi-structured interviews with social workers to validate results from the previous phases.

We developed interview guides for the semi-structured interviews and focus group interviews based on results from quantitative analyses, which are elaborated upon in Appendix A. These analyses were conducted using data from the Lifelines Cohort and Biobank Study, a large multidisciplinary cohort study in the northern Netherlands that examines health and health-related behaviors across multiple generations (34). The quantitative analysis applied mediation techniques, i.e., the Karlson-Holm-Breen (KHB) method (35), to examine the role of lifestyle and psychosocial factors in explaining socioeconomic inequalities in health outcomes metabolic syndrome (MetS) and major depressive disorder (MDD).

The interview guides were further based on the Integrated Model for Behavioural Change (i-Change model) (36), and on the Social Determinants of Health model (18). According to the i-Change model, behavior is determined by an individual’s motivation and intention. The model integrates the ideas of the Social Cognitive Theory, the Health Belief Model, Theory of Planned Behavior, the Trans-Theoretical Model, and Goal Setting Theory (37–41). The model proposes that a person’s behavior is determined by awareness, knowledge, attitudes, subjective norms, self-efficacy, and intention (36). According to the Social Determinants of Health Model, an individual’s behavior and health are determined by economic stability, education access and quality, health care access and quality, neighbourhood and built environment, and social and community context (18). The interview guide as used in phase one – for the policy professional and social workers in training – focused on awareness, motivation and intentions to have a healthy lifestyle (Appendix B).

The interview guide as used in phase two – for the health care professionals and policy professionals – also contained questions regarding approaches toward encouraging individuals with low SEP to adopt a healthy lifestyle (Appendix C). The interviews in phase three, with social workers, were aimed to deepen the information from the previous phases (Appendix D). To enhance rigor, we ensured consistency in data collection through a structured protocol, as well as an iterative process of data collection and analysis, implying that the findings are credible, reproducible, and not unduly influenced by individual interviewer bias. The results of the quantitative analysis informed the development of the interview guides by identifying key explanatory factors, such as education, health literacy, and social contacts in health disparities. The qualitative study was then used to explore the mechanisms behind these associations in more depth, allowing for a richer understanding of the lived experiences and perspectives of the target populations. Data collection was continued until data saturation was reached.

All focus group interviews lasted 2 h; the semi-structured interviews lasted 1 to 1.5 h and took online place via Microsoft Teams due to COVID restrictions, except for the focus group interview with social workers in training, which took place face-to-face. An experienced qualitative researcher (JAL, female health scientist [PhD]) moderated the focus group interviews, and a PhD student (LAH, female health scientist [MSc]) observed the focus group interviews and conducted the semi-structured interviews. Researchers and participants had not met before the interviews took place. Some participants knew each other beforehand. All interviews were conducted between October 2021 and February 2022.

### Data analysis

The interviews were audiotaped and transcribed verbatim. To ensure credibility, we maintained a reflexive logbook to document analytical decisions and enhance transparency. We used a thematic analysis approach as described by Braun and Clarke (42). To strengthen dependability and confirmability, we utilized investigator triangulation, with multiple researchers independently coding transcripts and discussing discrepancies. This process served as a form of cross-validation, ensuring that interpretations were not solely dependent on a single researcher’s perspective. First, two researchers (LAH and MAA, female health scientist [PhD]) read all transcripts individually and noted initial ideas. Second, we generated initial codes, performing systematic, line-by-line analysis of the transcripts, using Atlas.ti (version 9). Third, we organized these codes into preliminary themes related to influencing factors and approaches to decrease SEP lifestyle inequalities. Fourth, we reviewed themes in relation to the coded extracts and the entire data set, generating a ‘thematic map’. To ensure trustworthiness, we applied investigator triangulation by discussing emerging themes with multiple researchers involved in the study, incorporating diverse perspectives for cross-validation (42) To maximize dependability and reduce bias, we used an iterative team approach involving group discussion to group codes into themes (LAH, MAA, UB female health scientist [PhD], and MLAdK, female health scientist [MD, PhD]). Fifth, we refined and reviewed the initial coding framework and corresponding themes to ensure coding and theming were structured, and consistent with the research aims. Sixth, we reported and selected illustrative quotes, which were translated by a native speaker.

## Results

### Descriptive of the sample

The study included 28 professionals, with an average of 9.3 years of experience (Table 1). They were divided into four groups: social workers (11%), social workers in training (32%), health care professionals (25%), and policy professionals (32%). The average ages for these groups were 32.7, 17.0, 48.3, and 38.7, respectively. The majority of social workers and those in training were female (66.6%), while 88.9% of policy professionals and all health care professionals were female.

### Realizing a healthy lifestyle in individuals with low SEP

The analysis revealed five themes: (1) Individual factors, (2) Accumulating problems, (3) Social context (parents, peers, neighbours), (4) Societal factors (government), and (5) Involvement of low-SEP individuals. Themes 1–4 address both influencing factors of an unhealthy lifestyle and approaches for a healthy one, while theme 5 pertains solely to approaches. All quotations referenced in brackets (e.g., Q1.1, Q2.3) refer to Table 2.

**Table 2:** Main themes with exemplary quotes.

Main themes	Exemplary quotes
Individual factors	Q1.1: “*They (i.e., individuals with low SEP) are not crazy, you know. So I would say the basic level, and in my view the level to achieve sufficient health gains is there.*” (Policy professional, male, 37 years)
	Q1.2: “*If you are in a certain pattern, then it is hard to change it. And I also feel that people then often look for a cause outside themselves.*” (Health care professional, female, 33 years)
	Q1.3: “*They (i.e., individuals with low SEP) often do not have the discipline or the willpower to make something of their lives because they often choose the easier way, and therefore a lifestyle with unhealthy food.*” (Social worker, male, 25 years)
	Q1.4: “*I also think that they (i.e., individuals with low SEP) think mostly in the now. That they do not think about tomorrow or whatever or whenever.*” (Social worker, female, 19 years)
	Q1.5: “*In elementary school, a lot of things can also already be taught to the children; there is already a lot of profit to be made from that.*” (Health care professional, female, 53 years)
	Q1.6: “*A child was picked up from the schoolyard. Then that child said I have had fruit skewers, to which the parents said, did you have to eat fruit skewers? Then we will go to McDonalds tonight. Yeah, if that is the effect, well, in my opinion you should stop with the fruit skewers.*” (Policy professional, male, 37 years)
	Q1.7: “*It is not that those people (i.e., individuals with low SEP) do not get it. It is just that they are not receptive to the information, because they are in a different stage of life. So providing more information is not the solution to make sure those people have more knowledge.*” (Policy professional, male, 37 years)
	Q1.8: “*Then he sees such a professional once a month, or once every two months. He passes on information in one hour. And then the person has to work with that knowledge himself.*” (Policy professional, male, 37 years)
Accumulating problems	Q2.1: “*Often with low SEP there is not just one problem, but there are multiple problems, and people really experience permanent stress and therefore also lose the overview.*” (Health care professional, female, 59 years)
	Q2.2: “*Work, income, education. If those are not in order, as I see it there is no point in working on health promotion.*” (Policy professional, male, 37 years)
	Q2.3: “*A lot of worries, a lot of problems, whether it is about income or with children or whatever. And so the stress that comes with that also, well, as it were, paralyzes your brain, like then you are less able to think logically.*” (Health care professional, female, 43 years)
	Q2.4: “*Very often you have to start solving other problems before they are ready to start working on their weight, because you need energy and attention and time for that, too. Yeah, and if your energy and attention and time are on finding housing or stressing about your job or no job, then there is no room for it.*” (Health care professional, female, 59 years)
	Q2.5: “*When we are talking about people with low SEP who also already have, for example, accumulating problems and are running into all kinds of problems in terms of participation, health, you name it, then I think it is important that it be about providing perspective.*” (Policy professional, female, 31 years)
	Q2.6: “*You actually see almost in every project that based on “what does the resident himself want,” that you can make huge steps. That is also kind of naturally affiliated with positive health, is not it, where can someone be of value themselves or what does the person himself want.*” (Policy professional, female, 28 years)
	Q2.7: “*A broad approach to how you then solve a health problem. And that the solution does not have to take place only in the medical domain. But that you actually need a collaborative network of both healthcare and social professionals. And preferably also non-professionals from the environment where people live.*” (Health care professional, female, 43 years)
Social context	Q3.1: “*They (i.e., individuals with low SEP) are in a certain social network and then that goes from generation to generation and then you see these children as well, who are a bit bigger or a bit older, they deal with it in the same way.*” (Health care professional, female, 53 years)
	Q3.2: “*There is a lot of loneliness in that neighbourhood. A lot of people live alone. Well, then of course you start eating out of boredom. And then you start eating because you are not really hungry.*” (Policy professional, male, 37 years)
	Q3.3: “*Those children of highly educated parents, their parents say, here is five euros, go and see what you can buy at the supermarket, with what you have learned today. And the children of the low SEP target group, their parents say, yes, we are just going to do something normal. And that does not mean that those children just cannot do anything with that knowledge. But they (i.e., individuals with low SEP) also learn that this knowledge is not meant for them. So they grow up with the idea that this subject is not part of my environment or my world.*” (Policy professional, male, 37 years)
	Q3.4: “*Or let someone be invited too, because it often gets tricky when you say, like, that is there. But then you do not go there yet. Huh, but that someone is actually invited to be in a certain place at a certain time.*” (Social worker, female, 54 years)
Societal factors	Q4.1: “*In the Netherlands they basically say: Small government, self-initiative, self-direction. Yes, of course, that fosters that gap.*” (Policy professional, male, 37 years)
	Q4.2: “*But that school is responsible for children to get breakfast, no, as far as I am concerned …, that is part of parenting, I think.*” (Social worker, female, 54 years)
	Q4.3: “*You used to have the housekeeping school as a form of education. What happened there, cooking and handicrafts and, yes, a few housekeeping skills, that that should be allowed to come back a little bit.*” (Social worker, female, 54 years)
	Q4.4: “*We actually need to look more at how we can change society and how we can ensure that as a society it is less natural for there to be so many unhealthy things for sale. The difference in that, for example, fruit and vegetables, that that is just extremely expensive, while the unhealthy things are still extremely cheap.*” (Policy professional, female, 35 years)
	Q4.5: “*That is often a search, also in the direction of accountability of course, which you have to deal with as a municipality. Because we all know that prevention takes a long time to produce results, but, well, sometimes it is good to be able to demonstrate that.*” (Policy professional, female, 28 years)
Involvement of individuals with low SEP	Q5.1: “*I think that is also very individual. With some people it is better to apply shock therapy. Like: “This is not going well.” And with others, a bit more of a gentle hand. So I think it is very individual and what fits someone, what their character is. So this is also actually tailor-made.*” (Social worker, female, 54 years)
	Q5.2: “*I think that young adolescents, for example, do …, because they often want to do things differently than what you learn at home. So that might be an opportunity, like, to put in new information and knowledge.*” (Social worker, female, 54 years)
	Q5.3: “*One of the pillars of the approach is partial ownership, so that you really look together with the inhabitants of a neighbourhood, for example, to see what they need. And this also includes: how can you adapt these in such a way that they become usable and more effective for this group of people.*” (Health care professional, female, 43 years)
	Q5.4: “*But you have to ask, what do you think is good for you? What do you yourself think needs to change? Where do you want to start? And our idea is often different from their idea. And I think that is really the crux. Really genuinely listening to what he or she wants and thinks is important.*” (Social worker, female, 54 years)
	Q5.5: “*They (i.e., individuals with low SEP) can also get the knowledge from the internet, it is just about the guidance afterwards.*” (Policy professional, male, 37 years)

#### Theme 1: individual factors

##### Influencing factors

Participants emphasized various individual factors affecting the healthy lifestyle of low-SEP individuals, including awareness, knowledge, risk perception, habits, attitudes, self-efficacy, and skills. While low-SEP individuals possess basic knowledge about health and risks (Table 2, Q1.1), their attitudes toward a healthy lifestyle are often negative. They tend to prioritize immediate concerns over long-term health risks, like smoking and excessive drinking, which become ingrained habits (Q1.2). Many participants believed these individuals lacked determination, perseverance, self-efficacy, or discipline to adopt and maintain a healthy lifestyle (Q1.3). Additionally, these individuals often live day-to-day and have other priorities besides health risks (Q1.4).

##### Approaches

Regarding individual factors, participants stressed the need for education to enhance knowledge, awareness, risk perception, and self-efficacy in lifestyle choices. This education should cover healthy behavior and the risks of unhealthy choices, potentially starting in primary school (Q1.5). Some suggested beginning during adolescence when young individuals make their own choices. However, caution was advised against involving children in educating their parents, as it might lead to unhealthy compensatory behaviors (Q1.6).

Participants cautioned against assuming that adults with low SEP wish to become healthier or lack knowledge about healthy living. They advocated addressing other stressors like work, income, housing, and finances before promoting a healthy lifestyle. Emphasizing the consequences of an unhealthy lifestyle and the potential for change was deemed important (Q1.7).

Effective knowledge transfer to low-SEP individuals was emphasized. They often rely on others and social media for information. Brief, self-implemented knowledge transfer may not suffice; combining it with activities and repetition can help individuals apply knowledge and develop skills (Q1.8).

#### Theme 2: accumulating problems

##### Influencing factors

All participants acknowledged that various challenges hinder low-SEP individuals from leading healthy lives. These obstacles include low societal participation, unemployment, meagre income, poverty, addiction, mental health issues, and financial and housing-related stress (Q2.1). They stressed the importance of societal participation, which can provide income and a future outlook necessary for adopting a healthy lifestyle (Q2.2).

Poverty and financial stress negatively impact lifestyle choices, leading to avoidance of healthcare, smoking for relaxation, and purchasing less healthy food due to affordability (Q2.3). Furthermore, participants emphasized that parental poverty significantly affects children’s health, limiting their access to sports activities due to financial constraints (Q2.4). These issues often coexist and demand considerable effort to address, leaving individuals with little time or energy to improve their lifestyle.

##### Approaches

Participants stressed the need for an integrated approach to address the complex issues faced by low-SEP individuals, emphasizing coherence and cross-domain collaboration (Q2.5). They advocated prioritizing individuals’ life goals and providing a path to achieving them, aligning with the “positive health” approach (43) (Q2.6).

To tackle these accumulating problems, participants called for collaboration across different domains like social care and policy. Health care professionals should refer patients to social workers and local networks more frequently. They also recommended improving the knowledge of care providers about appropriate referrals for issues outside their domain. Government intervention to foster greater collaboration between domains was seen as essential for more impactful results (Q2.7).

#### Theme 3: social context

##### Influencing factors

Social influences shape an individual’s lifestyle from childhood. Parents play a pivotal role, positively or negatively impacting their children’s habits (Q3.1). As kids grow, peers become influential, sometimes positively, like warning against excessive alcohol or drug use.

In adulthood, partners have a significant impact on lifestyle. A negative view or the end of a relationship can lead to unhealthy behaviors like alcohol addiction (Q3.1). The social network in one’s neighbourhood or village is also crucial. It can combat loneliness, often linked to substance abuse (Q3.2).

##### Approaches

Participants stressed the importance of supporting parents in promoting a healthy lifestyle for their children. This could involve involving them in lifestyle-themed events at school or in the community centre. However, they cautioned against giving unsolicited advice, as it might backfire, causing parents to reject the health message (Q3.3).

To create a supportive social context for a healthy lifestyle, participants recommended connecting individuals through volunteer initiatives and role models within the community. Collaboration between the social, healthcare, and community sectors was seen as valuable. Additionally, organizers should actively invite individuals to neighbourhood activities (Q3.4).

#### Theme 4: societal factors

##### Influencing factors

Participants highlighted societal factors contributing to an unhealthy lifestyle among low-SEP individuals. In the Netherlands, policies emphasize individual initiative and responsibility, potentially widening inequality (Q4.1). Schools and professionals also influence lifestyles, although one participant, a social worker, believed healthy living should be the parents’ responsibility, not schools’ (Q4.2).

##### Approaches

Participants emphasized the societal responsibility to address the lifestyle and health of low-SEP individuals. They suggested several actions:

1. Assess available resources and best practices for improving their lifestyles (Q4.3).
2. Schools should regulate products, implement anti-smoking policies, provide information, model healthy behavior, and offer more physical activity opportunities (Q4.3).
3. Society should make healthy choices more accessible through measures like VAT reduction on fruits and vegetables and a “sugar tax.” Simplifying healthcare access is also crucial (Q4.4).
4. Municipalities should initiate efforts to reduce health disparities. However, participants noted challenges such as bureaucratic obstacles and limited financial resources (Q4.5).

#### Theme 5: involvement of individuals with low SEP

##### Approaches

Participants emphasized the importance of involving individuals in lifestyle change:

1. Tailor the message to each person, requiring professionals to be trained in working with low-SEP individuals (Q5.1).
2. Start involvement early, targeting adolescents who are receptive to change and breaking generational patterns (Q5.2).
3. Engage the target group in developing and implementing interventions to foster a sense of ownership (Q5.3).
4. Begin by understanding the individual’s priorities (Q5.4).
5. Ensure clear and understandable instructions in interventions, avoiding a pedantic approach. Allow more time for information processing and more frequent meetings (Q5.5).

## Discussion

Using the i-Change model (36) and the Social Determinants of Health model (18) we identified several themes regarding the realization of a healthy lifestyle in individuals with low SEP at both individual and societal levels: individual factors, accumulating problems, social context, societal factors, and involvement of individuals with low SEP. By integrating these theoretical models, we provide a structured understanding of the complex interplay between personal, social, and structural determinants of health.

### Individual factors

Our study shows that individuals with low SEP are influenced by various factors, including awareness, risk perception, self-efficacy, knowledge, attitudes, and habits, when it comes to leading a healthy lifestyle (9). This aligns with previous qualitative and quantitative research, which highlighted the impact of factors like lack of knowledge, risk perception, positive attitudes, negative personality traits, and low health literacy on their dietary and physical activity choices (44, 45).

Participants in our study, consistent with existing research, expressed concern regarding the motivation of individuals with low SEP to adopt a healthy lifestyle. They emphasized the importance of training professionals working with this population. This concern mirrors the broader sentiments of professionals working with disadvantaged communities (46). To address these issues, it is essential to move beyond individual behavioral approaches and incorporate structural interventions, such as regulatory policies on unhealthy food marketing, urban planning for accessible green spaces, or financial incentives for healthy lifestyles, that target social determinants of health (14). Such approaches can facilitate the connection between individual-level challenges, accumulating problems, and the social context. Furthermore, training should focus on tailoring healthy lifestyle messages to the specific needs of low SEP individuals (12, 22) and developing interventions that consider their knowledge, risk perception, and attitudes, allowing for more effective adaptation of interventions and health messages.

### Accumulating problems

Accumulating problems, like low income, poor housing, and unemployment, are significant barriers to healthy lifestyle adoption. This aligns with prior research, which highlighted similar challenges including high costs, financial stress, and time constraints (10, 12, 13). Participants in our study also noted poor housing and working conditions (47), along with chronic stress related to relationships, finances, and work, in line with quantitative findings (47, 48).

A key implication of our findings is the necessity for integrated, community-based interventions that address multiple accumulating problems simultaneously. Policies aimed at improving housing, financial security, and employment opportunities should be seen as essential components of lifestyle interventions rather than separate domains. Participants emphasized an integrated approach involving collaboration between social, policy-making, and healthcare domains to address accumulating problems (49). This aligns with the concept of ‘positive health’ that encourages adaptability and self-management when working with individuals with low SEP (43).

### Social context

The home environment, peers, partners, and neighbourhood significantly influence lifestyle habits and promotion in individuals with low SEP (9, 13). Qualitative studies have highlighted the positive impact of the social context on supporting a healthy lifestyle (10, 11, 27). However, a quantitative study revealed that low SEP individuals often experience isolation, emphasizing the importance of involving the neighbourhood’s social network (50).

Addressing the social context, it’s important to educate low SEP parents to encourage healthy lifestyles for their children (25, 26). Active parental involvement and considering parents’ personalities are key. Additionally, addressing isolation, a common issue among low SEP individuals, is essential (50). Community-driven interventions, including social support groups and peer mentorship programs, could enhance social cohesion and facilitate healthier behaviors in low SEP populations.

### Societal factors

Participants highlighted societal barriers, including the lack of initiative, responsibility, and direction, contributing to unhealthy lifestyles among low SEP individuals. This aligns with prior research (9, 51). According to participants, government and policymakers should assume a more significant role in supporting low SEP individuals. In particular, urban health equity must be prioritized, ensuring that individuals in low SEP communities have equal access to green spaces, affordable healthy food options, low exposures to pollution, and safe recreational areas. For example, the Dutch government could take the lead in organizing care to promote lifestyle changes and implementing policies to encourage a healthy lifestyle (52). While existing policies on healthy lifestyles are in place, they are considered inadequate (53). Our findings suggest that health-promoting policies should extend beyond healthcare settings to include urban planning, education, and social services, fostering a comprehensive approach to health equity.

### Involvement of individuals with low SEP

Participants emphasized the importance of tailored, well-considered health messages, early involvement, and personal guidance. This aligns with prior research, highlighting the need for comprehensive, culturally sensitive support, prioritizing goal-setting over mere information provision (12, 22, 23). Co-creation of interventions with individuals from low SEP backgrounds is important to ensure their relevance and effectiveness.

### Strengths and limitations

A major strength of this study is that we explored not only factors influencing lifestyle choices among groups with low SEP, but also approaches by professionals closely involved with the target population for replacing unhealthy lifestyles with healthy ones. The credibility of the findings was enhanced by using an iterative process, different qualitative methods, and the use of a team approach for analysing the results. We were able to identify multidisciplinary perspectives by including different professionals, thereby increasing the robustness and transferability of the results. Furthermore, we reached data saturation, indicating that all major aspects are likely to have been identified. Our study also has some limitations. We cannot rule out the possibility of selection bias, as only the most interested participants may have participated in our study. These participants may also have been professionals very committed to the well-being of the individuals with low SEP, possibly feeling sorry for the individuals and making the issues worse. Information bias may also have occurred, with participants, themselves coming from a low SEP household, speaking from their own experience, and not just from that of the individuals they are currently working with.

### Implications

Our results have practical, policy, and government implications. We’ve identified factors and approaches to enhance lifestyles for low SEP individuals from a multidisciplinary perspective. Collaboration among professionals in healthcare, policy-making, and social domains is crucial. In particular, our study highlights the need for stronger intersectoral policies that integrate health and social care services to create a more supportive environment for individuals with low SEP. Governments should allocate more resources to address fundamental low SEP issues, using measures like sugar taxes, VAT reductions on healthy foods, and anti-smoking zones. Cross-domain expertise can streamline the resolution of accumulating problems. Further research should prioritize quantitative approaches to assess the impact of previously unstudied factors, such as government regulations, on reducing SEP health inequalities. Moreover, while this study was in the Netherlands, its generalizability may vary in different contexts.

## Conclusion

To our best knowledge this study is the first on the professionals’ views regarding lifestyle promotion in individuals with low SEP. Our findings indicate that professionals emphasize the need for a positive, integrated approach in addressing the accumulation of problems in this population. Additionally, the findings highlight the necessity for more elaborate governmental initiatives at both the individual and societal levels to support the adoption of a healthy lifestyle among individuals with low SEP. Moreover, individuals with low SEP should be involved more intensively in the development of interventions. Our findings also underscore the importance of urban health equity, particularly access to health-promoting environments, as a key priority for future interventions and policy measures. This study demonstrates that multiple factors must be addressed simultaneously to ensure the success of a healthy lifestyle intervention or strategy. Further research should explore the effectiveness of specific policy measures and intervention strategies to improve health outcomes in this population.

## Data Availability

The original contributions presented in the study are included in the article/Supplementary material, further inquiries can be directed to the corresponding author.
