# Supplementary material for: Healthy lifestyle in low-socioeconomic groups: expert views
Source: Front Public Health. 2025 May 21;13:1511317. doi: 10.3389/fpubh.2025.1511317 (PMC12133870; doi:10.3389/fpubh.2025.1511317)
Supplement: Supplementary file 1 [file Data_Sheet_1.pdf]

SUPPLEMENTARY MATERIAL

Appendix A. Summary of the results of the quantitative analyses.

Appendix B. Interview guide for interviews with social workers (in training), phase 1.

Appendix C. Interview guide for interviews with policy and health care professionals, phase 2.

Appendix D. Interview guide for interviews with social workers (in training), phase 3.

Supplementary Tables

Table S1. COREQ (COnsolidated criteria for REporting Qualitative research) checklist.

Table S2. Multivariable mediation analysis of the most important mediating factors in the association between SEP and incident and remitted metabolic syndrome, using the Karlson-Holm-Breen method.

Table S3. Multivariable mediation analysis of the most important mediating factors in the association between SEP and incident and remitted major depressive disorder, using the Karlson-Holm-Breen method.

Appendix A. Summary of the results of the quantitative analyses.

Main results of the quantitative study were that: 1) compared to household equivalized income and occupational prestige, education is the most important SEP determinant of the development and remission of both MetS and MDD; 2) educational differences in the development and remittance of MetS in the quantitative study were explained mainly by diet, smoking, alcohol use and health literacy (Supplementary Table 2); and 3) educational differences in the development and remittance of MDD were explained mainly by quality of social contacts, by smoking, and by health literacy (Supplementary Table 3).

Appendix B. Interview guide for semi-structured interview with policy professionals and focus group interview with social workers in training, phase 1.

Awareness

What do you know about a healthy lifestyle?

- Knowledge: What do you see as dangers/risks of unhealthy behavior?
- Information: What kind of health information do you read or hear?
  - Personal factors: Do you understand the information you receive about health? What is and what isn't healthy?
  - Message factors: When someone tells you to change some habits (e.g., quit smoking or cut back on alcohol consumption, or eat healthier), what do you think?
  - Channel factors: Where do you get health information? How and where would you like to get it?
  - Source factors: From whom do you get information about health? Who would you like to get health information from?
- Risk perception: What do you know about the risks associated with unhealthy behavior?
- Insight: What kind of healthy habits do you have? And what kind of unhealthy habits?

Motivation

What do you think of a healthy lifestyle?

- Attitude: How important do you think it is to live a healthy life and, for example, to stop smoking or drink less alcohol?
- Social influence: How important do your family and friends think it is to live a healthy life?

- Self-efficacy: What do you do to maintain healthy behavior?

Intention

How would you approach a healthy lifestyle?

- Plan to action/execute: What habits do you now have for living a healthy life? And what else do you need to stop smoking, drink less alcohol, or eat healthier (focused on participants, if they indicate that they want to stop smoking, for example)? Who or what can help you to quit smoking or eat healthier?
- Skills: What else do you need to be able to do to stop smoking, drink less alcohol, or eat healthier?
- Barriers: Why is it (sometimes) impossible to quit smoking/alcohol use/unhealthy eating? (focused on participants)

Appendix C. Interview guide for interviews with policy and health care professionals, phase 2.

Following a presentation on the quantitative results:

- Which findings of the project do you recognize in your work? What is new?
- Which factors should be addressed with preventive interventions? At what ages?
- Which factors do you think deserve priority?
- Which interventions aimed at these factors do you know or apply?
- For which factors are there as yet no suitable interventions?

Following presentation on qualitative results of focus groups in low SEP groups:

- What type of interventions do you think of after this explanation?
- What type of interventions should be developed?
- How do you make interventions effective for people with a low SEP? For example, what adjustments need to be made?
  - Information factors: How do you think you can effectively communicate information about a healthy lifestyle?
  - Personal factors: Making it understandable for the low SEP target group.
  - Message factors: Conditions of the message.
  - Channel Factors: Channel of information. TV/newspaper/social media/folder, in general practice/oral by doctor.
  - Source Factors: Source of the information. A doctor/role model from the own group/parent/teacher.
- What tools do you need to be able to respond to these results?

- For which type of interventions are practical policy adjustments necessary? And what adjustments?
- How would you adapt interventions for people with low SEP?

Appendix D. Interview guide for interviews with social workers, phase 3.

Barriers/problems:

- What is currently going well within this target group when it comes to health and lifestyle?
- What problems do you see in this target group that can influence their failure to learn or maintain a healthy lifestyle?
    - How should this be addressed?

Accumulating problems is a frequently heard term when it comes to this target group. The target group has problems in various areas of life that prevent healthy living: a number of these are participation in society, loneliness, health problems, stress about finances, stress about work, stress about housing, etc.

- How do these problems influence each other and what is their relationship with lifestyle and health?
- How should these problems be addressed?
    - Who should tackle these problems?

Knowledge:

- What knowledge about lifestyle and health does this target group have?
- How do you see the role of increasing knowledge to achieve a healthy lifestyle among this target group?

Related to knowledge; In our previous research, education level emerged as the most important factor among the various social and economic factors affecting health and a healthy lifestyle. This would imply that to promote a healthy lifestyle, education level should be considered first, and only then income or occupation.

- How do you see the role of education level for health and a healthy lifestyle? And how do you see the roles of income and occupation?

Information:

- What kind of information about healthy lifestyle and health does the target group read or hear?
    - Who is bringing this message?
    - Where is this message delivered?
    - Do they understand this?
    - What do they think of this?

- How can we best reach the target group with a health message?
    - What can be done to adapt the message of a healthy lifestyle to the target group?
    - What should be the purpose of the message?
    - What should be the content of the message?
    - Who can best deliver the message?
    - Where is this message best delivered?
    - What needs to be done to make it understandable?
    - What needs to be done to actually apply nurturing to the lifestyle of the target group?

Social influence parents:

- How do you see the role of parents in influencing the lifestyle of children and young people in this target group?
- Why do you think that the role of parents can be both positive and negative?
    - What are specific moments when the influence is positive? And moments when the influence is actually negative?
- What can parents do to support children with a healthy lifestyle?
    - How can parents be helped with this?

Social influence school:

- How do you see the role of the school in influencing the lifestyle of children and young people in this target group?
- Why do you think the role of the school can be both positive and negative?
    - What are specific moments when the influence is positive? And moments when the influence is actually negative?
- What can schools do to support children with a healthy lifestyle?
    - How can schools be helped with this?
- When should we start with prevention?

Self-efficacy:

- What is the target group already doing to learn healthy behavior and to be able to maintain it?

- And where do you see it going wrong?

- What is needed [from professionals, for example] to teach the target group healthy behavior and have it maintained?
- Who should help the target group to learn and maintain healthy behavior?
- At what point should the target group be helped to learn and maintain healthy behavior?

Interventions:

- Which interventions do you know that are tailored to this target group to tackle lifestyle? [for each intervention mentioned ask what works and what does not work]
    - Which elements/parts work well within these interventions?
    - Which elements/parts do not work well within these interventions?
    - What should such an intervention contain to make it effective for the target group?
        - People with a low level of education?
        - People with a low income/in poverty?
        - People with low occupational prestige/without a job?
    - What is the best timing or age to apply such an intervention?

Closing question:

- What is your most important advice or tip for us if we want to develop an intervention for this target group?

Supplementary Tables.

Table S1. COREQ (COnsolidated criteria for REporting Qualitative research) checklist.

**Topic**	**Item No.**	**Guide questions/description**	**Reported on page no.**
Domain 1: research team and reflexivity			
Personal characteristics			
Interviewer/facilitator	1	Which author/s conducted the interview or focus group?	Data collection section
Credentials	2	What were the researcher’s credentials? E.g. PhD, MD	Data collection section
Occupation	3	What was their occupation at the time of the study?	Data collection section
Gender	4	Was the researcher male or female?	Data collection section
Experience and training	5	What experience or training did the researcher have?	Data collection section
Relationship with participants			
Relationship established	6	Was a relationship established prior to study commencement?	Participants section

Topic	Item No.	Guide questions/description	Reported on page no.
Participant knowledge of the interviewer	7	What did the participants know about the researcher? e.g. personal goals, reasons for doing the research	Participants section
Interviewer characteristics	8	What characteristics were reported about the inter viewer/facilitator? e.g. Bias, assumptions, reasons, and interests in the research topic	Participants section
Domain 2: Study design			
Theoretical framework			
Methodological orientation and Theory	9	What methodological orientation was stated to underpin the study? e.g. grounded theory, discourse analysis, ethnography, phenomenology, content analysis	Data analysis section
Participant selection			
Sampling	10	How were participants selected? e.g. purposive, convenience, consecutive, snowball	Participants section
Method of approach	11	How were participants approached? e.g. face-to-face, telephone, mail, email	Participants section

**Topic**	**Item No.**	**Guide questions/description**	**Reported on page no.**
Sample size	12	How many participants were in the study?	Participants section
Non-participation	13	How many people refused to participate or dropped out? Reasons?	Participants section
Setting			
Setting of data collection	14	Where were the data collected? e.g. home, clinic, workplace	Data collection section
Presence of non-participants	15	Was anyone else present besides the participants and researchers?	Data collection section
Description of sample	16	What are the important characteristics of the sample? e.g. demographic data, date	Results section
Data collection			
Interview guide	17	Were questions, prompts, guides provided by the authors? Was it pilot tested?	Participants section
Repeat interviews	18	Were repeat interviews carried out? If so, how many?	Participants section

Topic	Item No.	Guide questions/description	Reported on page no.
Audio/visual recording	19	Did the research use audio or visual recording to collect the data?	Data analysis section
Field notes	20	Were field notes made during and/or after the interview or focus group?	Data analysis section
Duration	21	What was the duration of the interviews or focus group?	Data collection section
Data saturation	22	Was data saturation discussed?	Data collection section
Transcripts returned	23	Were transcripts returned to participants for comment and/or correction?	Data collection section
Domain 3: analysis and findings			
Data analysis			
Number of data coders	24	How many data coders coded the data?	Data analysis section
Description of the coding tree	25	Did authors provide a description of the coding tree?	Data analysis section
Derivation of themes	26	Were themes identified in advance or derived from the data?	Data analysis section
Software	27	What software, if applicable, was used to manage the data?	Data analysis section

Topic	Item No.	Guide questions/description	Reported on page no.
Participant checking	28	Did participants provide feedback on the findings?	Data analysis section
Reporting			
Quotations presented	29	Were participant quotations presented to illustrate the themes/findings? Was each quotation identified? e.g. participant number	Results section
Data and findings consistent	30	Was there consistency between the data presented and the findings?	Results section
Clarity of major themes	31	Were major themes clearly presented in the findings?	Results section
Clarity of minor themes	32	Is there a description of diverse cases or discussion of minor themes?	Results section

Table S2. Multivariable mediation analysis of the most important mediating factors in the association between SEP and incident and remitted metabolic syndrome, using the Karlson-Holm-Breen method.

	**Education**	**Income**	**Occupational prestige**
	**Percentage mediated**	**Percentage mediated**	**Percentage mediated**
**Incidence**			
Smoking	8.4	-8.8	4.9
Diet quality	3.6	5.3	2.2
Alcohol intake	3.6	24.6	5.2
Health literacy	5.5	8.1	4.2
**Remittance**			
Smoking	3.2	0.9	9.4
Diet quality	4.7	2.3	10.5
Alcohol intake	3.5	10.1	15.1
Health literacy	4.4	2.1	7.3

OR: odds ratio; CI: confidence interval; analyses were controlled for years of education, household equivalent income, occupational prestige, age, and sex at baseline, and time between baseline and the second assessment; * $P<0.05$.

Table S3. Multivariable mediation analysis of the most important mediating factors in the association between SEP and incident and remitted major depressive disorder, using the Karlson-Holm-Breen method.

	**Education**	**Income**	**Occupational prestige**
	**Percentage mediated**	**Percentage mediated**	**Percentage mediated**
**Incidence**			
Social participation	11.1	17.7	28.1
Smoking	12.1	-2.4	9.5
Health literacy	14.1	5.3	13.8
**Remittance**			
Social participation	5.9	12.2	56.8
Smoking	-1.8	8.1	18.5
Health literacy	16.2	5.6	23.3

OR: odds ratio; CI: confidence interval; analyses were controlled for years of education, household equivalent income, occupational prestige, age, and sex at baseline, and time between baseline and the second assessment; * P<0.05.
